# Non-O1 *Vibrio cholerae *inguinal skin and soft tissue infection with bullous skin lesions in a patient with a penis squamous cell carcinoma

**DOI:** 10.1186/1476-0711-8-17

**Published:** 2009-05-19

**Authors:** Aitziber Aguinaga, María E Portillo, Jose R Yuste, Jose L del Pozo, Emilio García-Tutor, Jose L Pérez-Gracia, José Leiva

**Affiliations:** 1 Department of Clinical Microbiology, University Hospital of Navarra, Pamplona, Spain; 2 Infectious Diseases Division, University Hospital of Navarra, Pamplona, Spain; 3 Department of Plastic and Reconstructive Surgery, University Hospital of Navarra, Pamplona, Spain; 4 Department of Medical Oncology, University Hospital of Navarra, Pamplona, Spain

## Abstract

*Vibrio spp*. is a pathogen rarely isolated in cancer patients, and in most cases it is associated with haematological diseases. Cutaneous manifestations of this organism are even rarer. We report a case of Non-O1 *Vibrio cholerae *inguinal skin and soft tissue infection presenting bullous skin lesions in a young type II diabetic patient with a penis squamous cell carcinoma having a seawater exposure history.

## Introduction

*Vibrio cholerae *is mainly related to water sources [1]. Contaminated seawater exposure or contaminated seafood ingestion are frequently associated with diarrhoea and/or extraintestinal infections such as otitis media, skin and soft tissue infections (SSTI) and bacteremia [1,2]. *Vibrio spp*. is a pathogen rarely isolated in cancer patients, and in most cases it is associated with haematological diseases. Although the clinical picture may have a wide range in *Vibrio spp*. SSTI, bullous lesions are almost exclusively associated with *V. vulnificus *infection [3,4] and have rarely been reported with non-O1 *V. cholerae *infections [4,5].

We report here the case of a non-O1 *V. cholerae *SSTI presenting bullous skin lesions in a diabetic patient with a solid tumour.

## Case report

A 36-year-old patient from the Canary Islands (Spain) with controlled type II diabetes mellitus was diagnosed with a moderately differentiated squamous cell carcinoma of the penis in December 2006 in his local hospital. The patient underwent a partial penectomy. In the initial follow-up after surgery, the abdominal CT and granulation tissue were normal. The patient had been exposed to seawater and seafood from December 2006 to October 2007 when bullous skin lesions were observed in both inguinal regions during a examination. Lesions were fitted with inguinal metastases, and the patient underwent inguinal surgery followed by radiotherapy.

**Figure 1:**
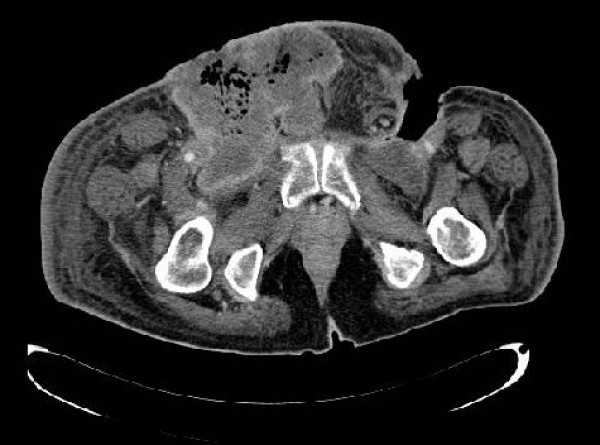
**Several large necrotic adenopathies in inguinal, scrotal and pubic area (CT scan)**.

In November 2007 he was admitted to our hospital for a second consultation. Physical examination showed stinking lesions in both inguinal regions with cellulitis and bullae. Patient underwent an abdominal computed tomography (CT) scan with contrast that revealed the existence of several large necrotic adenopathies in the inguinal, scrotal and pubic areas (Figure 1). A right abdominal wall nodule and a left groin abscess extending from psoas muscle to skin were also observed. A CT scan of the chest showed pulmonary lesions compatible with metastases. Initial laboratory findings showed increased a white blood cell count of 20,3 × 10^9^/L with 88,1% neutrophils, haemoglobin of 8,3 g/dL and a C-reactive protein of 8,7 mg/dL (normal range 0–1 mg/dL).

**Figure 2:**
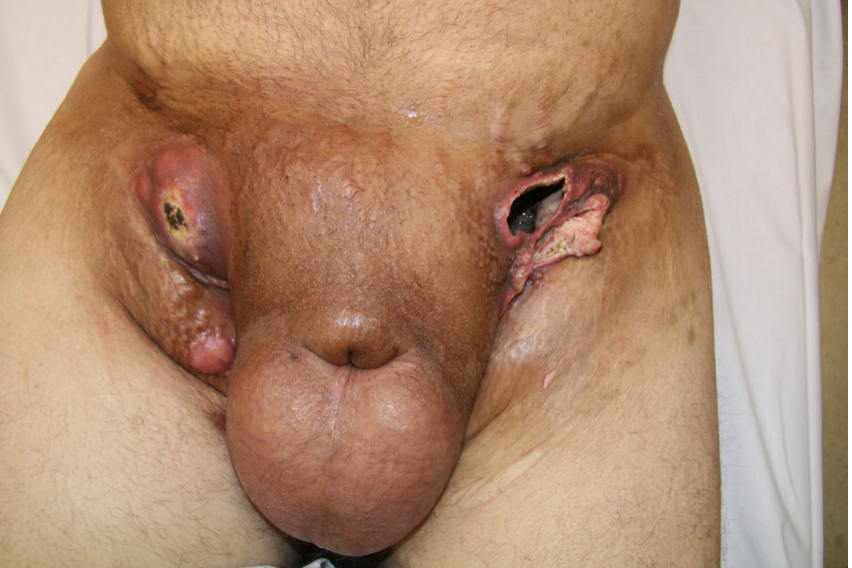
**Skin lesions and cellulitis after a mayor cure**.

The patient was treated with a left inguinal region debridement, percutaneous drainage and flamazine therapy. Purulent fluid was obtained by swabbing from the fistulized left bulla and was sent for culture (Figure 2). At that time, two sets of blood cultures were drawn. Empiric intravenous antimicrobial therapy with ceftriaxone 1 g/24 h and metronidazole 500 mg/8 h was started. Daily cures with Flamazine were also indicated. A Gram stain of the sample showed abundant polymorphonuclear leukocytes, and cultures yielded *Escherichia coli, Serratia marcescens and V. cholerae *susceptible to the empiric antimicrobial therapy instituted. Blood cultures were sterile after incubation for six days. As the patient was not improving after 7 days of therapy, a new sample was taken with a syringe. Methicillin-susceptible *Staphylococcus aureus *and the same *E. coli*, characterized by phenotypic methods (biotype and antimicrobial susceptibility) were isolated. Those microorganisms were carbapenems-susceptible so the antibiotic therapy was switched to ertapenem 1 g/24 h i.v. and cellulitis gradually improved.

A new sample was taken for control with a syringe from the left inguinal lesion after 2 weeks of treatment with ertapenem. Enriched alkaline peptone water (1% NaCl pH 8.5) and TCBS agar were added to the conventional media plates and only *V. cholerae *was again isolated. The therapy was changed to oral levofloxacin 500 mg/12 h. After one month of treatment with levofloxacin a new sample for control from the left inguinal lesion was taken with a syringe, resulting sterile after incubation for a week in selective media. Thus, the levofloxacin dosage was switched to oral levofloxacin 500 mg/24 h. After 40 days with levofloxacin 500 mg/24 h, the right bulla fistulized, so a new debridement and percutaneous drainage was done in order to eliminate the necrotic tissue. New samples of both inguinal regions were obtained by means of syringe and were found sterile after 7 days of incubation in selective media.

During this time, the patient was treated with two cycles of first- and second-line palliative chemotherapy and one cycle of third-line palliative chemotherapy. Due to the metastasis progression, it was decided to stop the chemotherapy. In March, the patient returned to the Canary Islands under treatment with oral levofloxacin 500 mg/24 h. The patient died two weeks later due to his underlying disease.

*V. cholerae *is a curved Gram-negative rod that grows as beta-hemolytic mucous colonies on blood agar plates and yellow colonies on thiosulfate-citrate-bile salts-sucrose (TCBS) agar plates. *V. cholerae *was identified by two commercial identification systems: VITEK 2 (bioMérieux^® ^SA, Marcy-L'Etoile, France) and API 20E *Enterobacteriaceae *(bioMérieux^®^, France). It was susceptible to the O/129 vibriostatic agent (Oxoid, Ltd., Basingstoke, UK), and slide agglutination tests with polyvalent antisera showed a non-O1, non-O139 serotype. Antibiotic susceptibility testing was performed by standard disk diffusion method on Mueller-Hinton agar, and minimal inhibition concentrations (MICs) were determined by the Etest^® ^diffusion method (AB Biodisk^®^, Solna, Sweden). According to CLSI guidelines interpretative criteria for *Vibrio spp*. [6] the strain was susceptible to ampicillin (8 μg/ml), amoxicillin-clavulanic (4 μg/ml), ceftriaxone (0.016 μg/ml), ceftazidime (0.25 μg/ml), gentamicin (1 μg/ml), tobramycin (4 μg/ml), amikacin (8 μg/ml), ciprofloxacin (0.004 μg/ml), levofloxacin (0.008 μg/ml), piperacillin-tazobactam (0.38 μg/ml), chloramphenicol (8 μg/ml), trimethoprim-sulfamethoxazole (0.125 μg/ml) and doxycycline (2 μg/ml). The sample was sent to the National Center for Microbiology (Majadahonda, Madrid, Spain) and was confirmed to be non-O1 *V. cholerae *non-producer of toxin. The gene *sodB *for the identification of *V. cholerae *was detected. Neither the genes *wbeO *and *rfb *for the O1 and O139 serotype identification, nor the gene *ctxA *encoding cholera toxin were detected by multiplex PCR technique.

## Discussion

SSTI are often deep and devastating [7]. Etiology may be mono or polymicrobial involving a mixed aerobe-anaerobe bacterial flora [7]. Although this case involved a necrotizing skin and soft-tissue infection with polymicrobial etiology repeated *V. cholerae *isolates suggested *V. cholerae *was likely a major player in this patient's infection.

*Vibrio spp*. SSTI may range from bullous skin lesions and localised cellulitis to severe necrotizing soft-tissue infection with secondary septicaemia [8,9]. *V. vulnificus *followed by *V. parahaemolyticus *and *V. alginolyticus *are the species most commonly isolated from wounds whereas non-O1 *V. cholerae *is the less common involved species [3,10]. Although bullous lesions occur mainly in patients with *V. vulnificus*, infection, bullae in non-O1 *V. cholerae *infection have been rarely reported [4,5].

*Vibrio spp*. infections are rarely documented in cancer patients and they are infrequent in solid tumours [11]. Cellulitis due to non-O1 *V. cholerae *is rare and it is normally associated with the presence of chronic underlying diseases such as liver cirrhosis, diabetes mellitus, immunocompromised states or haematological malignancies such as chronic lymphocytic leukaemia, acute myeloid or lymphoblastic leukaemia and multiple myeloma or lymphocytic lymphoma [2,4,5,11-17].

To the best of our knowledge, only 13 cases of non-O1 *V. cholerae *extraintestinal infections in immunocompromised patients have been reported since 1978 (Table 1). Only one case had a documented cellulitis [12]. Patients were largely male (10 cases) with a mean age of 61 years (range, 36–78 years). Risk factors for *Vibrio spp*. infection were found in ten cases; 8 of them had liver disease. Interestingly, 10 patients had a solid tumour and only 3 patients had a hematological disease. Twelve cases presented fever, bacteremia or diarrhoea. Nine patients survived. The remaining patients died from *V. cholerae *infections or from underlying diseases. Our case is remarkable because it represents the second reported case of bullous lesions and cellulitis associated with non O1-*V. cholerae *in a cancer patient.

**Table 1:** Clinical summary of 14 published cases of non-O1 *V. cholerae *extraintestinal infections in immunocompromised patients.

Age (years)/gender	Clinical syndrome	Risk factors other than neoplasia	Underlying condition	Outcome	Reference
72/m	Fever Bacteremia	Renal insufficiency	MM	Recovered	[13]
51/m	Pneumonia, diarrhoea	-	CLL	Expired	[14]
59/m	Bacteremia	Cirrhosis	Hepatoma	Recovered	[1]
69/m	Ascites	Cirrhosis	Hepatoma	Expired	[1]
50/m	Bacteremia	Cirrhosis	Hepatoma	Recovered	[1]
54/m	Bacteremia	Cirrhosis	Hepatoma	Recovered	[1]
60/f	Diarrhoea	Cirrhosis	Hepatoma	Recovered	[15]
36/m	Bacteremia	Cirrhosis	Hepatoma	Expired	[15]
78/m	Fever Bacteremia	-	NSCLC	Recovered	[11]
62/m	Cachexia Fever	Liver insufficiency	GC	Recovered	[16]
77/m	Fever Splenic abscess	Diabetes	PC	Expired	[17]
78/f	Ascites Bacteremia	Chronic liver disease	CC	Recovered	[12]
54/f	Bacteremia Cellulitis	No	ML	Expired	[5]
36/m	Cellulitis	Diabetes	SCPC	Expired	Reported case

MM, multiple myeloma; CLL, chronic lymphocytic leukemia; NSCLC, non-small-cell lung carcinoma; GC, gastric cancer; PC pancreatic cancer; CC, cervix carcinoma; ML, myeloid leukemia; SCPC, squamous-cell penis carcinoma.

The case reported shows that non O1-*V. cholerae *may produce wound infections in cancer patients with infrequent clinical manifestations. In conventional mediums *Vibrio spp*. can easily remain undetected especially when polymicrobial infections occur. Consequently, *Vibrio spp*. should be considered in the differential diagnosis of any SSTI in immunocompromised patients, principally those occurring after seafood ingestion or contact with salt or estuary water. Thus, in these cases, physicians should alert the microbiology laboratory to add selective and enriched culture mediums in addition to routine media.

## Consent

Written informed consent was obtained from the patient for publication of this case report and accompanying images. A copy of the written consent is available for review by the Editor-in-Chief of this journal.

## Competing interests

The authors declare that they have no competing interests.

## Authors' contributions

AA and MP carried out the microbiological assays and drafted the manuscript. JY and JdP participated in clinical infectious disease's diagnosis and treatment. EGT carried out inguinal debridement. JPG participated in the chemotherapy. JL participated in its design and coordination. All authors read and approved the final manuscript.
